# Potential Off-Target Interaction of the Amyloid PET Imaging Tracer PiB with Acetylcholinesterase

**DOI:** 10.1021/acsomega.5c06188

**Published:** 2025-10-10

**Authors:** Alberto Granzotto, Rosa Fullone, Ludovico Miccoli, Manuela Bomba, Claudia Di Marzio, Stefano Delli Pizzi, Giuseppe Floresta, Stefano L. Sensi

**Affiliations:** † Center for Advanced Studies and Technology − CAST, 9301 University G. d’Annunzio of Chieti-Pescara, via dei Vestini 31, Chieti 66100, Italy; ‡ Department of Neuroscience, Imaging, and Clinical Sciences, University G. d’Annunzio of Chieti-Pescara, via Luigi Polacchi 11, Chieti 66100, Italy; § Institute of Neurology, SS Annunziata University Hospital, University G. d’Annunzio of Chieti-Pescara, via dei Vestini 5, Chieti 66100, Italy; ∥ Institute for Advanced Biomedical Technologies − ITAB, University G. d’Annunzio of Chieti-Pescara, via Luigi Polacchi 11, Chieti 66100, Italy; ⊥ Department of Drug and Health Sciences, 9298 University of Catania, viale Andrea Doria 6, Catania 95125, Italy; # Psychopharmacology, Drug Misuse and Novel Psychoactive Substances Research Unit, School of Life and Medical Sciences, University of Hertfordshire, Hatfield AL10 9AB, U.K.

## Abstract

Pittsburgh compound B (PiB) is a widely used Positron Emission Tomography (PET) tracer for detecting amyloid-β (Aβ) deposits in Alzheimer’s disease (AD). While PiB is assumed to bind selectively to Aβ, emerging evidence suggests off-target interactions that may complicate PET signal interpretation. Here, we report that PiB can interact with acetylcholinesterase (AChE), a key enzyme in the cholinergic system. Similarity screening identified the AChE ligand thioflavin T (ThT) as the top structural analogue of PiB. Docking studies and molecular dynamics simulations showed that PiB stably binds the peripheral anionic site (PAS) of AChE, with binding energies comparable to ThT and clinically relevant AChE inhibitors. *In vitro* fluorescence-based assays confirmed this interaction and suggest an involvement of the PAS. These findings indicate a plausible, stable off-target interaction between PiB and AChE with implications for interpreting PiB-PET signals in AD, particularly in reference regions with altered AChE expression or under AChE inhibitor therapy.

## Graphical abstract

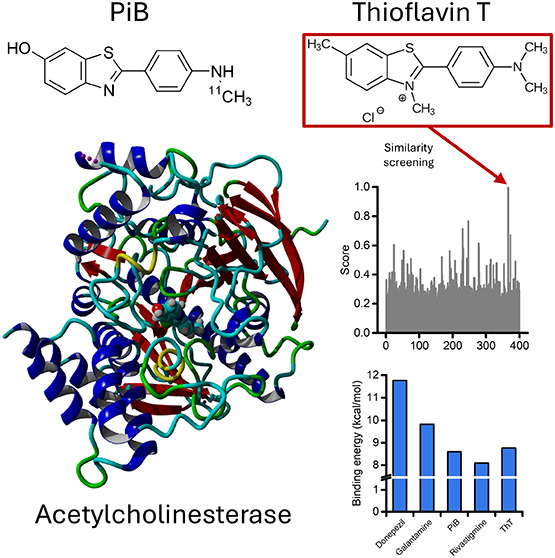

## Introduction

Positron emission tomography (PET)-based biomarkers are largely employed in research and clinical settings for disease diagnosis and monitoring, patient stratification, or as an efficacy outcome of interventions.^1^ In Alzheimer’s disease (AD), PET tracers have been developed to quantitatively detect changes in the accumulation of key pathological markers, like cortical amyloid-β (Aβ) deposits, hyperphosphorylated tau (p-tau) protein buildup, and neurodegeneration.^2,3^ Alterations in these biomarkers mirror disease progression and are the “gold standard” for diagnosing AD and for the early detection of people at risk of developing the condition.^4,5^ The shift from a clinical- to a biomarker-based definition of AD is also at the basis of the “ATN research framework”, a biological definition of the disease (i.e., “A” – amyloid, “T” – tau, and “N” – neurodegeneration) aimed at offering a quantifiable and unbiased staging of AD.^4^ The approach is relevant since the pathological alterations of AD can occur and are detectable long before the onset of cognitive and behavioral symptoms.^6,7^ Early identification of individuals in the very early stages of the condition represents a transformative step in the effective development and targeted implementation of disease-modifying interventions.

Alterations of Aβ levels are widely recognized as one of the earliest molecular changes that can foreshadow the onset of AD pathology, although the specific contribution of Aβ to disease pathogenesis is debated.^8,9^ Quantitative assessment of Aβ is performed either in biological fluids like liquor and plasma, where decreases in Aβ abundance reflect the cerebral deposition of the peptide, or by PET-based imaging, where specific radioligands are employed to detect the presence of fibrillar Aβ aggregates in the brain. Several Aβ radiotracers have been developed since the early 2000s, with Pittsburgh compound B (^11^C-PiB), a thioflavin T (ThT) analogue, being the first of this class of imaging agents. The short half-life of ^11^C-PiB led to the development of fluorine-18 derivatives more suitable for clinical applications, like ^18^F-flutemetamol or the *trans*-stilbene-based compounds ^18^F-florbetapir and ^18^F-florbetaben. Nevertheless, ^11^C-PiB is still broadly adopted in clinical research settings.

Although these radioligands are widely employed for the diagnosis of AD and for monitoring target engagement of Aβ-targeting interventions, doubts have been cast on their specificity and sensitivity.^10–13^ Previous studies demonstrated that 2-aryl-6-hydroxybenzothiazole-based tracers can effectively bind to off-target molecules, like sulfotransferases, that likely contribute to PET signals unrelated to the overall Aβ load.^10,12^ However, it is unclear whether this class of Aβ radioligands has additional off-target effects.

This study aims to investigate PiB binding characteristics at the molecular level, and by employing unbiased *in silico* screening, docking calculations, molecular dynamics (MD) simulations, and *in vitro* assay, we surveyed for potential novel binding partners unrelated to Aβ pathology.

## Results and Discussion

To identify potential off-target partners of Aβ PET tracers, we performed an unbiased screening of biologically relevant molecules that show structural similarity to PiB by employing the SwissSimilarity 2021 Web Tool. Our analysis returned the score of 400 molecules (Figure 1A and Table S1) with, as expected, ThT being the top-scoring molecule (score 0.996).^14,15^ More importantly, ThT was identified because the molecule is a ligand for acetylcholinesterase (AChE) in the Protein Data Bank (PDB ID: pdb_00002j3q). To test the hypothesis that PiB interacts with AChE, we performed docking studies within the AChE pocket using the crystal structure of human AChE (PDB ID: pdb_00004ey7). AChE has two binding sites: the catalytic site and the peripheral anionic site (PAS).^16,17^ We focused on the latter, located at the entrance of the catalytic gorge, since it mediates the interaction of AChE with ThT.^18–20^ Analysis of docking results shows that PiB has a binding energy comparable with that of ThT (8.603 and 8.771 kcal/mol, respectively; Figure 1B). The binding energy of clinically approved AChE inhibitors (donepezil, galantamine, and rivastigmine) was calculated for comparison (Figure 1B). Figure 1C,D shows the two- and three-dimensional poses and the interaction of PiB with the amino acid residues in the AChE PAS. PiB forms a π–π-sulfur interaction with residue Phe297 along with several hydrophobic, alkyl, and van der Waals interactions with residues Trp86, His447, Tyr337, Phe338, Tyr72, Trp286, Phe295, Tyr341, and Tyr124 (Figure 1C,D).

**Figure 1:**
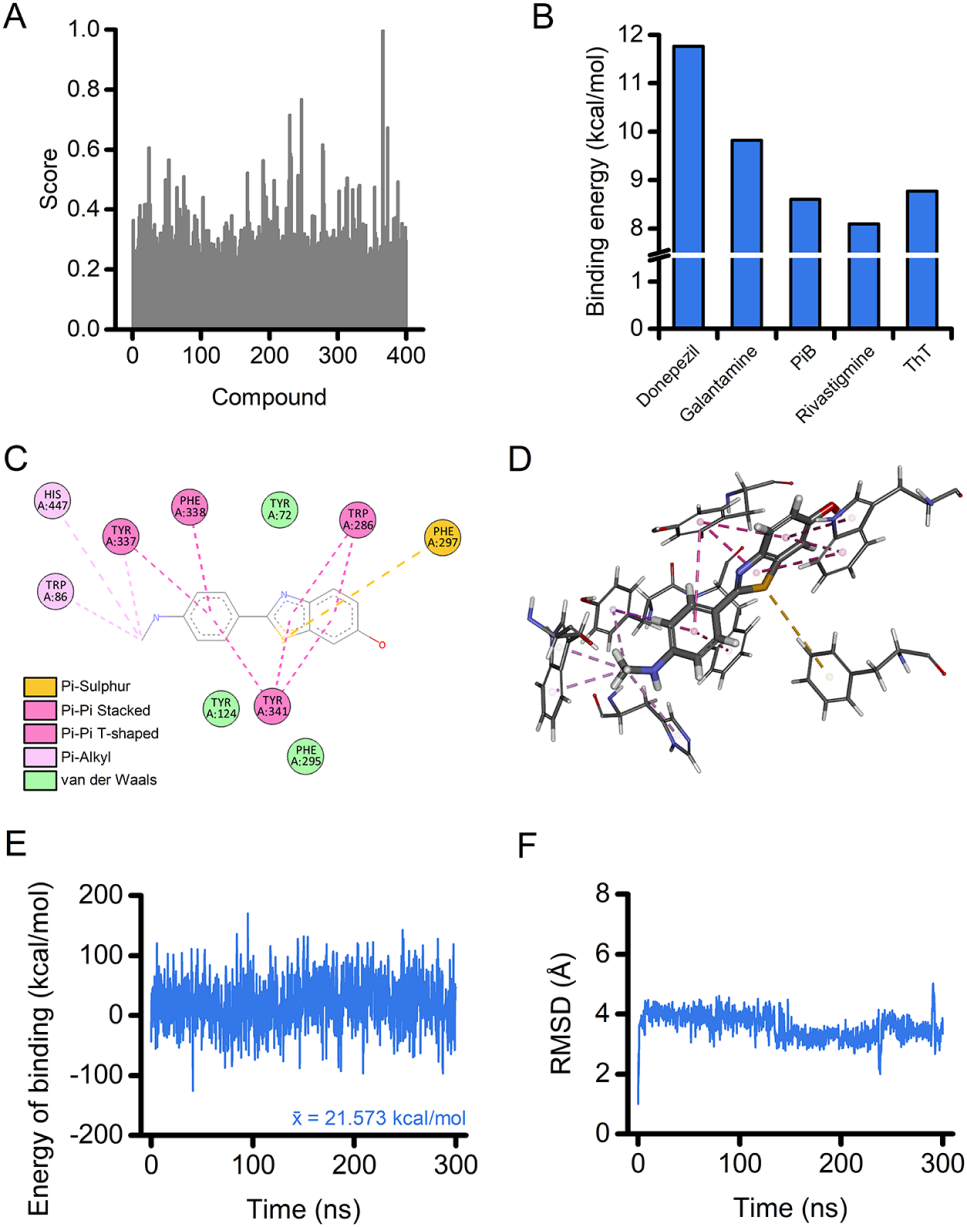
Identification and *in silico* characterization of AChE as a potential target of PiB. (A) The plot illustrates the similarity score of each compound screened with the SwissSimilarity 2021 Web Tool. (B) The histogram depicts the binding energy calculation of the listed ligands after docking on AChE. (C-D) Two-dimensional (C) and three-dimensional (D) docking poses and interactions of PiB in the AChE PAS. The dashed yellow line indicates π–π-sulfur interaction; dashed pink lines indicate π–π-alkyl interactions; magenta lines indicate π–π and T-shaped interactions; green residues show van der Waals interactions. (E, F) Time course of energy of binding (E) and root-mean-squared displacement (RMSD; F) for the PiB-AChE complex over a 300 ns MD simulation.

We further investigated the PiB-AChE complex by performing a 300 ns molecular dynamics (MD) simulation. Analysis of the energy of binding shows that PiB maintains a high and stable binding energy throughout the simulation (Figure 1E). The stability of the PiB-AChE complex is also supported by the root-mean-square deviation (RMSD) analysis of the ligand movement after superimposing the molecule on the enzyme structure (Figure 1F). After a stabilization phase, the ligand remains within the AChE PAS. The compound exhibits only a few modest, sharp fluctuations that return to baseline levels during the simulation (Figure 1F). We attribute the stability of the complex to the sulfur interaction, along with the dense network of hydrophobic interactions that keep PiB within the AChE PAS.

To determine whether PiB forms a stable complex with AChE *in vitro*, we leveraged the intrinsic spectroscopic properties of the compound. The molecule displays an absorbance and an emission maximum at 348 and 432 nm, respectively (Figure 2A). We measured the fluorescence signal of PiB after incubation with or without AChE from *Electrophorus electricus* (eeAChE), followed by size-exclusion filtration to remove unbound ligand. Samples incubated with eeAChE retained a significantly higher fluorescence signal when compared with the PiB sample alone, indicating PiB binding to the enzyme (Figure 2B,C). The specificity of this approach was confirmed by performing a similar set of experiments in which PiB was incubated with bovine catalase, an enzyme that shares with eeAChE a tetrameric structure and a similar molecular weight (Figure 2D,E).

**Figure 2:**
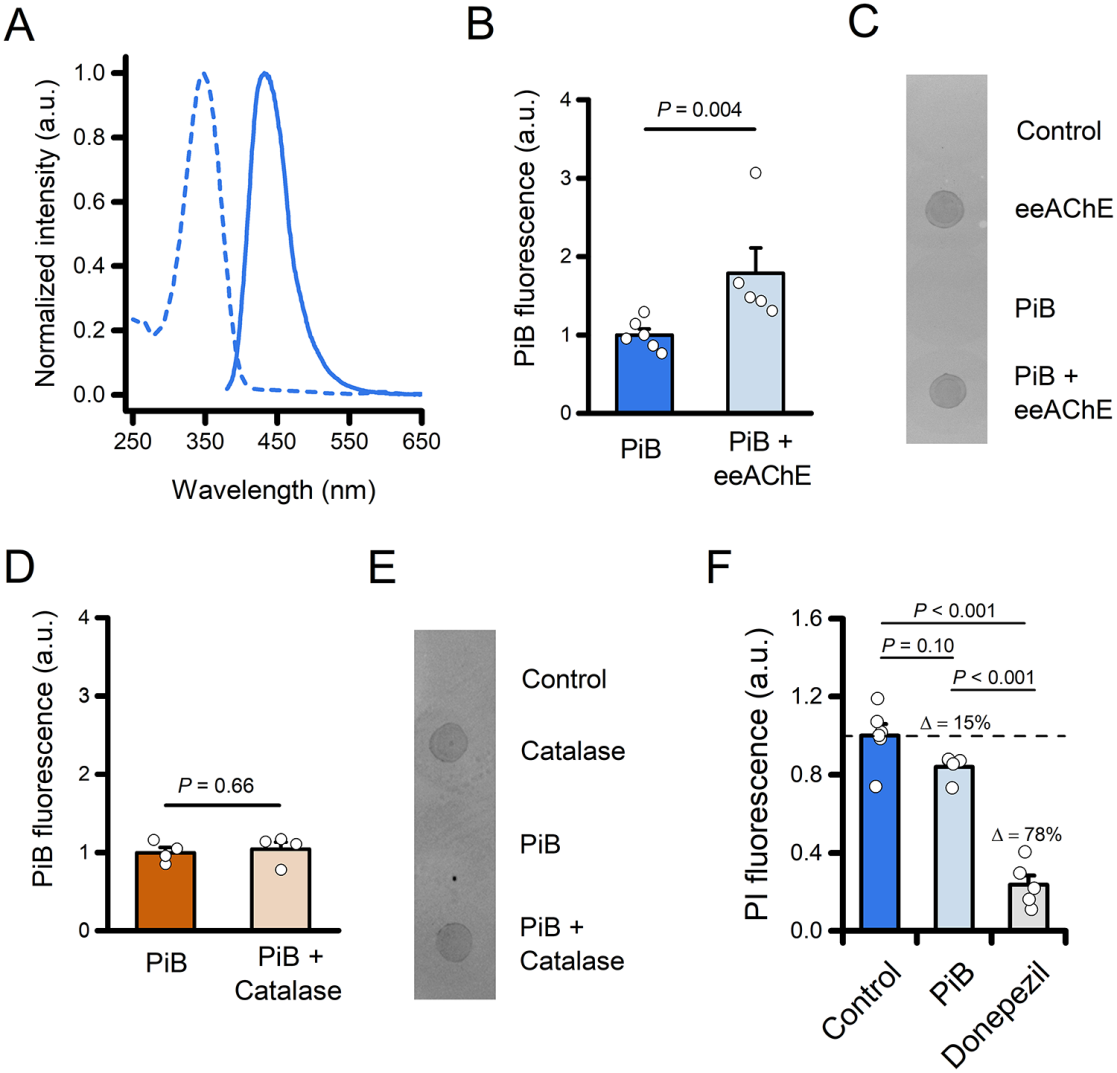
Experimental validation of the formation of the PiB-eeAChE complex. (A) Absorption (dashed line) and emission (solid line) normalized spectra of PiB in a KPi buffer (pH 7.4). (B) The bar graph depicts normalized fluorescence of PiB following incubation of the compound with or without eeAChE (2 μg) and size-exclusion filtration (PiB *n* = 6 and PiB + eeAChE *n* = 5 independent experiments). (C) Ponceau S staining of the retentate was spotted onto a nitrocellulose membrane to assess protein recovery. (D) The bar graph depicts normalized fluorescence of PiB following incubation of the compound with or without catalase (2 μg) and size-exclusion filtration (PiB *n* = 4 and PiB + Catalase *n* = 4 independent experiments). (E) Ponceau S staining of the retentate was spotted onto a nitrocellulose membrane to assess protein recovery. (F) The bar graph depicts normalized fluorescence of PI following incubation with eeAChE in the presence of vehicle (Control; 0.8% DMSO), PiB (20 μM, 0.8% DMSO), or Donepezil (20 μM, 0.8% DMSO). Note the ≈15% signal reduction in the presence of PiB. In panels (B and D), the comparison of mean values was assessed by the Mann–Whitney U Test. In panel (F), mean values were compared by one-way ANOVA followed by Tukey’s post hoc test.

To further evaluate PiB binding to the PAS of eeAChE, we performed propidium iodide (PI) displacement, an assay used for probing the interaction of candidate drugs with the PAS of AChE.^21^ The binding of PI to the PAS increases dye fluorescence; meanwhile, its displacement by PAS-interacting compounds leads to signal reduction.^22^ In the presence of 20 μM PiB, PI fluorescence was reduced by approximately 15% compared with control conditions (Figure 2F). Although this difference did not reach statistical significance (*P* = 0.10), the data suggest a potential trend toward PI displacement. The reduction increased to ≈78% in the presence of the high-affinity, PAS-binding AChE inhibitor donepezil (20 μM; Figure 2F).^16,23^

Together, these findings support the formation of a stable PiB–eeAChE complex *in vitro* and are consistent with the possibility that PiB interacts with the PAS of the enzyme.

In this study, we provide computational and experimental evidence that the amyloid PET tracer PiB can bind AChE, suggesting a previously unrecognized off-target interaction. Our findings extend prior observations that ThT-based compounds may interact with nonamyloid targets and raise important questions on the specificity of PiB and related PET tracers used in AD research and diagnostics.^18^

Our *in silico* similarity screening identified ThT, a known AChE ligand, as the compound most structurally related to PiB among biologically relevant molecules in the PDB, pointing to a potential interaction between PiB and AChE. We further tested this hypothesis using molecular docking and MD simulations. Docking results showed that PiB binds the PAS of AChE with binding energy comparable to ThT and within the range of clinically relevant AChE inhibitors. PiB establishes π–sulfur and hydrophobic interactions with residues located in the PAS and near the gorge of the active site, like Phe297, Trp286, Tyr337, and His447. These residues were found to be key for the interaction with AChE-targeting drugs.^16^ MD simulations further confirmed the persistence of these interactions, indicating a stable and energetically favorable complex.

We validated the computational predictions with a binding assay that exploits the intrinsic fluorescence properties of PiB, supporting the formation of a stable PiB–AChE complex *in vitro* and suggesting that the interaction occurs at the PAS of the enzyme.

We acknowledge that without a direct determination of the dissociation constant (*K*_d_), the affinity of PiB for AChE remains uncertain. However, a more thorough investigation of the interaction between PiB and AChE, such as the dissociation constant (*K*_d_), was hampered by both the physicochemical properties of PiB and the sensitivity of AChE to PiB-compatible solvents. We found that in conditions suitable for the AChE enzymatic assay, PiB began to precipitate at concentrations above 25 μM (Figure S1B). Also, the use of alternative solvents or surfactants was unsuccessful (unpublished observations). Moreover, the use of higher concentrations of DMSO substantially impairs AChE activity.^24^ Thus, our current in vitro data support the occurrence of binding but do not allow us to quantify the affinity. In a further attempt to directly examine PiB-PAS interaction, we also tested an *in vitro* competition assay in the presence of donepezil.^16,23^ However, preliminary control experiments showed a substantial spectral overlap between PiB and donepezil (Figure S1B), making fluorescence-based comparisons unfeasible. While these technical constraints limit our ability to perform orthogonal or competitive binding assays, they do not undermine the core observation that PiB interacts with AChE, as suggested in our fluorescence-based filtration assay and PI displacement.

The identification of AChE as a potential off-target of PiB has several implications. First, it imposes the need to carefully interpret PET signals in brain regions where AChE is abundantly expressed, particularly in early-stage or atypical AD presentations, where Aβ deposition may not be the unique contributor to the tracer uptake. Second, given that AChE expression and activity can change in the aging brain and neurodegenerative conditions beyond AD,^25,26^ the off-target binding of PiB to AChE could contribute to false positives or elevated baseline/background signals in specific populations. Third, the PiB signal could be influenced by the use of AChE inhibitors that act by binding the PAS of the enzyme.

Our results do not challenge the widely accepted view that PiB primarily targets fibrillar Aβ. Rather, they offer a mechanistic explanation that could contribute, alongside other factors, to some of the known limitations in amyloid PET imaging, like the substantial overlap in cortical standardized uptake value ratios (SUVRs) observed among cognitively healthy older adults, individuals with mild cognitive impairment, and AD patients.^12^ During PET imaging, brain concentrations of [^11^C]­PiB are typically in the nanomolar range, several orders of magnitude lower than the micromolar concentrations used in our *in vitro* assay. Consequently, if the PiB–AChE interaction has a micromolar *K*_d_, its contribution to PET binding is likely to be modest. Nonetheless, the relatively high density of AChE in the human brain—estimated around 0.6 μg of AChE per gram of tissue in neocortex and higher in striatum and cerebellum^27^—suggests that even low-affinity binding could, in principle, contribute to background signal in specific brain areas. Simply put, a weak, nonspecific interaction between PiB and AChE in amyloid-poor regions—like the cerebellum, an AChE-rich area^28^ commonly used as a reference for PET quantification—could subtly influence SUVR calculations and reduce the apparent contrast between groups. This effect may be particularly relevant considering the brain-wide decline in AChE levels observed along the AD continuum.^29^

Our findings align with previous reports of off-target binding for other radiotracers used in AD, for which interactions with enzymes like monoamine oxidases and sulfotransferases have been reported.^10,11,30^ In addition, our similarity virtual screening does not rule out the presence of additional yet untested PiB binding partners. Finally, the high lipophilicity of PiB could also explain the elevated retention of the tracer in lipid-enriched white matter regions.^31,32^

To our knowledge, this is the first study to suggest a plausible interaction between PiB and AChE at both the computational and experimental levels. While the actual contribution to PET signals *in vivo* remains to be established, such interactions may affect PiB signals and data interpretation. Further studies using radiolabeled PiB and AChE inhibitors *in vivo* are warranted to confirm whether this interaction occurs under pathophysiological conditions and contributes to PET signals.

In conclusion, these results underscore the importance of integrative approaches combining computational modeling with biochemical validation to uncover and assess the biological relevance of such interactions.

## Methods

### Reagents and Chemicals

PiB was purchased from TargetMol; eeAChE, catalase from bovine liver, and all of the other chemicals were from Sigma-Aldrich.

### Library Screening

Similarity screening for the PiB amyloid PET tracer was performed with the SwissSimilarity 2021 Web Tool (http://www.swisssimilarity.ch/ ^33,34^ ) on August 3, 2024. The search was limited to ligands present in the Protein Data Bank (LigandExpo; 19500 compounds) using a consensus 2*D*/3D screening using a score based on both FP2 Tanimoto coefficient and Electroshape-5D Manhattan distance.^33^ Screening scores and SMILES notations were downloaded for further analysis.

### Molecular Modeling

All of the molecules investigated in this study were downloaded as three-dimensional conformer.sdf files from PubChem.^35^ The Energy Minimization Experiment function, using YASARA AutoSMILES for the automatic force field parameter assignment, was used to optimize the 3D structure before docking.

From the PDB (PDB ID: pdb_00004ey7), a cell encompassing all atoms extending 5 Å from the surface of the structure of the ligand was generated, and the crystallized ligand was removed. Global ligand docking was performed using VINA using the default parameters and further refined with VINA Local Search.^37^

The molecular dynamics simulations of the acetylcholinesterase complexes were run with the same YASARA suite^38^ by employing the macro *md_runfast*. A cuboid periodic simulation cell extending 20 Å from the protein surface was set and filled with water (density: 0.997 g/mL). The setup included an optimization of the hydrogen bonding network^39^ to increase the solute stability, and a p*K*_a_ prediction to fine-tune the protonation states of protein residues at pH 7.4.^40^ NaCl ions were added at a physiological concentration of 0.9%. After steepest descent and simulated annealing minimizations to remove clashes, the simulation was run for 300 ns using the AMBER14 force field^41^ for the solute, GAFF2 ^42^ and AM1BCC^43^ for ligands, and TIP3P for water. The cutoff was 8 Å for van der Waals forces,^44^ and no cutoff was applied to electrostatic forces (using the Particle Mesh Ewald algorithm).^45^ The equations of motions were integrated with a multiple time step of 2.5 fs for bonded interactions and 5.0 fs for nonbonded interactions at a temperature of 298 K and a pressure of 1 atm (NPT ensemble) using algorithms described previously.^46^ MD conformations were recorded every 250 ps. The energies of binding and the MD trajectory have been calculated using the *md_analyzebindenergy* macro implemented in the YASARA suite employing the MM/PBSA method as previously described.^36,47^ Ligand movement RMSD was calculated with the YASARA *md_analyze* function after superposition on the receptor.

### PiB Spectra

A 20 μM PiB (0.8% DMSO final concentration) solution was prepared in 100 mM potassium phosphate buffer (KPi, pH 7.4). Absorbance spectrum was measured by employing a PerkinElmer Lambda 35 spectrophotometer (Range: 200–900 nm; slit: 2 nm; resolution: 1 nm; speed: 240 nm/min). Fluorescence emission spectrum was measured with a BioTek Synergy H1 plate reader (Ex λ: 350 nm; Em range: 380–700 nm; resolution: 1 nm; gain: 60 au).

### Turbidity Assay

The turbidity assay was performed as previously described.^48^ In brief, the absorbance of increasing concentrations of PiB (1.56 to 1600 μM) was measured at 405 nm using a PerkinElmer SPECTRAmax 190 microplate reader. Absorbance readings from the buffer alone (KPi containing 0.8% DMSO) served as a reference. The purpose of the assay was to determine the highest PiB concentration that does not result in precipitation of the compound.

### Fluorescence-Based Interaction Assay

A fluorescence-based binding assay was performed to assess the potential interaction between PiB and eeAChE. PiB (25 μM final concentration; dissolved in 100 mM KPi, 0.1% DMSO) was incubated *in vitro* either in the presence or absence of 2 μg of eeAChE in a total volume of 50 μL. Incubations were carried out for 5 h at 30 °C under gentle agitation (1100 rpm). Following incubation, each mixture was filtered using Amicon Ultra-0.5 centrifugal filters with a 30 kDa molecular weight cutoff (Millipore), centrifuged at 14,000*g* for 10 min at room temperature to separate unbound PiB from AChE-bound PiB. The retentate, containing eeAChE and any bound PiB, was recovered and transferred to a black walled 96-well plate for fluorescence measurement. Fluorescence was measured using a BioTek Synergy H1 plate reader with excitation at 350 nm and emission at 440 nm. The retentate was subsequently spotted onto a nitrocellulose membrane, stained with Ponceau S, and imaged to assess protein recovery.

### Propidium Iodide Displacement Assay

To evaluate the interaction between PiB and the PAS of eeAChE, we employed a PI displacement assay. A total of 25 U of eeAChE were incubated overnight with PI (1 μM), either alone or in the presence of PiB (20 μM, 0.8% DMSO). A parallel experiment using donepezil (20 μM, 0.8% DMSO) served as the positive control. PI fluorescence was measured using a BioTek Synergy H1 plate reader with an excitation at 535 nm and emission at 630 nm. Background fluorescence from PI alone was subtracted from all of the readings. Data were then normalized as *F*_*x*_/*F*_vehicle_, where *F*_*x*_ represents the PI fluorescence for each condition, and *F*_vehicle_ is the PI fluorescence in the presence of eeAChE and 0.8% DMSO.

### Statistical Analysis

Microsoft Excel (Microsoft) and OriginPro 2025b (OriginLab) were employed for the statistical analysis and data plotting. Data in Figure 2 are represented as mean ± 1 standard error of the mean (s.e.m.); data points represent individual experiments. Exact *P* values are reported for each relevant comparison. The number of replicates and the statistical test used are provided in the figure legends.

## Supplementary Material



## Notes

Supporting Information contains PiB solubility data, the spectral properties of PiB and donepezil, and the table containing the small molecules identified by virtual similarity screening (PDF)

## Abbreviations

Aβ: amyloid-β
AChE: acetylcholinesterase
AD: Alzheimer’s disease
DMSO: dimethyl sulfoxide
eeAChE: *Electrophorus electricus* acetylcholinesterase
KPi: potassium phosphate buffer
PAS: peripheral anionic site
PDB: Protein Data Bank
PET: Positron Emission Tomography
PI: propidium iodide
PiB: Pittsburgh compound B
RMSD: root-mean-square displacement
SUVRs: standardized uptake value ratios
ThT: thioflavin T
