# Supplementary material for: Potential Off-Target Interaction of the Amyloid PET Imaging Tracer PiB with Acetylcholinesterase
Source: ACS Omega. 2025 Oct 10;10(41):48544–50. doi: 10.1021/acsomega.5c06188 (PMC12547588; doi:10.1021/acsomega.5c06188)
Supplement: Supplementary file 1 [file ao5c06188_si_001.pdf]

Potential off-target interaction of the amyloid PET imaging tracer PiB with acetylcholinesterase

Alberto Granzotto [1,2,*], *Rosa Fullone* [1,2], *Ludovico Miccoli* [1,2,3], *Manuela Bomba* [1,2], *Claudia Di Marzio* [1], *Stefano Delli Pizzi* [2,4], *Giuseppe Floresta* [5,6], *Stefano L. Sensi* [1,2,3,4]

[1] Center for Advanced Studies and Technology – CAST, University G. d'Annunzio of Chieti-Pescara, via dei Vestini 31, 66100, Chieti, Italy

[2] Department of Neuroscience, Imaging, and Clinical Sciences, University G. d'Annunzio of Chieti-Pescara, via Luigi Polacchi 11, 66100, Chieti, Italy

[3] Institute of Neurology, SS Annunziata University Hospital, University G. d'Annunzio of Chieti-Pescara, via dei Vestini 5, 66100, Chieti, Italy

[4] Institute for Advanced Biomedical Technologies – ITAB, University G. d'Annunzio of Chieti-Pescara, via Luigi Polacchi 11, 66100, Chieti, Italy

[5] Department of Drug and Health Sciences, University of Catania, viale Andrea Doria 6, 95125, Catania, Italy

[6] Psychopharmacology, Drug Misuse and Novel Psychoactive Substances Research Unit, School of Life and Medical Sciences, University of Hertfordshire, AL10 9AB, Hatfield, United Kingdom

Supporting Information

Figure S1

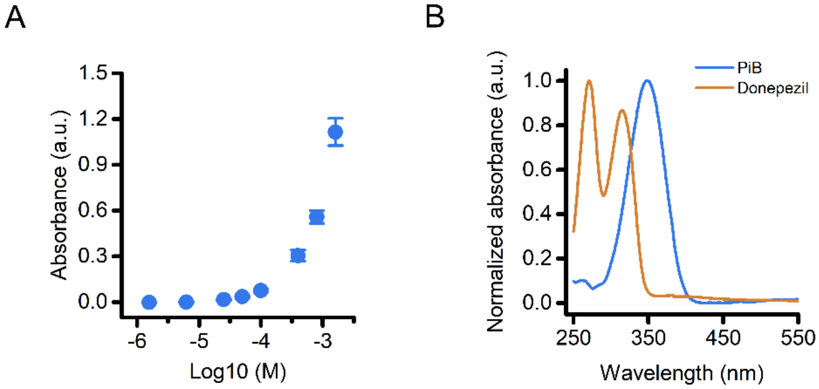

Limited PiB solubility and spectral properties of PiB and donepezil complicate fluorescence- and enzymatic-based assays. (A) The scatter plot depicts absorbance of PiB measured across increasing concentrations (1.56 – 1600 µM) and reveals precipitation above 25 µM, limiting its use in assays requiring higher concentrations. (B) Normalized absorbance spectra of PiB (blue) and donepezil (orange) highlight substantial spectral overlap which complicates interpretation of fluorescence-based competition assays involving both compounds. Data are representative of at least two independent experiments. a.u., arbitrary units.

Table S1

Ligand PDB ID	Similarity Score	SMILES
**TFL**	0.996	CN(C)C1=CC=C(C=C1)C1=[N+](C)C2=CC=C(O)C=C2S1
**GK1**	0.767	CC1=CC=C(O)C=C1NC1=CC=NC2=CC(=CC=C12)C1=CSC(C=O)=N1
**EVW**	0.715	NC1=NC2=CC=C(C=C2S1)C(=O)NC(C1=CC=CC=C1)C1=CC=CC=C1
**UUL**	0.672	OC1=CC=C(NC2=NC(=CS2)C2=CC=C(Cl)C=C2)C=C1
**JMT**	0.616	OC1=CC=C2NC=C(CN3CCN(CC3)C3=NC4=CC=CC=C4S3)C2=C1
**09H**	0.606	O=C(NC1=CC=CC=C1N1CCNCC1)C1=CSC(=N1)C1=CC=C2OCCC2=C1
**JMW**	0.594	OC1=CC=C2NC=C(CN3CCN(CC3)C3=NC4=CC(Cl)=CC=C4S3)C2=C1
**EWK**	0.585	NC1=NC2=CC=C(SCC3=CC=C(C=C3)C(=O)NCC3=CC=CC=C3)C=C2S1
**EWT**	0.574	NC1=NC2=CC=C(C=C2S1)C(=O)NCC1=CC(Cl)=C(Cl)C=C1
**2WJ**	0.565	CC(=O)NC1=NC2=CC=C(C=C2S1)C1=CC=CN=C1
**AQE**	0.563	C1CC[C@@]2(CCCN(C2)C2=C3C(NC=C3C3=NC=CS3)=NC=C2)NC1
**94U**	0.522	CCN(CC)C1=CC=C(NC(=O)C2=CC3=C(N2)N=CS3)C=C1
**G4A**	0.514	CN1\\C(OC2=CC=CC=C12)=C\\C1=[N+](CCCS(O)(=O)=O)C2=CC=CC=C2S1
**3TI**	0.51	OC1=CC=C(C=C1)\\N=C\\C1=C2C=CC=CC2=CC=C1O

**N0E**	0.505	OC1=CC=C(NC(=O)CCC2=CC=CC=C2)C=C1
**2JR**	0.5	C1CCN(C1)C1(CCCCC1)C1=CN=C(S1)C1=CC=C2NC=CC2=C1
**EV8**	0.498	COC(=O)C1CCN(CC1)C(=O)C1=CC=C(CNC(=O)C2=CC=C3N=C(N)SC3=C2)C=C1
**CP9**	0.497	CC1=NC2=CN=CC=C2N1C1=CC=C(CN2C(=O)SC3=CC=CC=C23)C=C1
**X1H**	0.492	COC1=CC=C(C=C1)C(=O)C1=C(SC2=CC(O)=CC=C12)C1=CC=C(O)C=C1
**B4K**	0.484	CC(=O)NC1=C2C=CC(=NC2=NN1)C1=CC=C(O)C(O)=C1
**P2X**	0.48	CC(C)N1N=C(C2=CC3=CC(O)=CC=C3N2)C2=C(N)N=CN=C12
**RU5**	0.475	NC(=O)C1=CC=C2NC(=NC2=C1)C1=CC=C(OC2=CC=C(Cl)C=C2)C=C1
**3F4**	0.474	OC1=CC=C(C=C1)C1=NC(=O)C2=CC=CC=C2N1
**MKY**	0.472	CCOC(=O)CN1\\C(SC2=CC(O)=CC=C12)=N\\C(N)=N
**OFI**	0.471	CCCC(=O)NC1=NNC2=CC(=CC=C12)C1=CC=C(O)C=C1
**NU3**	0.466	NC(=O)C1=C2N=C(NC2=CC=C1)C1=CC=C(O)C=C1
**LNJ**	0.462	COC1=CC=C(CC2=CC=C(C=C2)C2=CSC(N)=N2)C=C1
**C2J**	0.442	OC1=CC=C(C=C1)C1=CC=C2C(NN=C2NC(=O)C2CC2)=C1
**57X**	0.44	CCCN1C2=NNC(C3=CN=C(S3)C3=CC=CN=C3)=C2C=CC1=O
**972**	0.419	CC(C)COC1=CC=CC(C2=NC3=CC(C(N)=N)=C(Cl)C=C3N2)=C1O
**JV5**	0.418	CC1=CC=CC=C1OCC(=O)NC1=CC2=NNN=C2C=C1
**917**	0.417	CC(=O)NC[C@H]1CN(C(=O)O1)C1=CC=C(C=C1)C1=CN=CS1

**2RE**	0.416	OC1=CC=C(C=C1)C1=NC(=C(N1)C1=CC=NC=C1)C1=CC=C(F)C=C1
**0HD**	0.416	O=C(NCCC1=CC=CC=C1)NC1=CC2=CNN=C2C=C1
**656**	0.413	CC(C)COC1=CC=CC(C2=NC3=CC(=CC=C3N2)C(N)=N)=C1O
**3U6**	0.411	NC1=NN=C(S1)C1=CC=C2NC=C(C2=C1)C1=NC(NC2CCCC2)=CC=C1
**D58**	0.408	C[C@H]1NCCC[C@@H]1NC1=C2C=C(SC2=C(C=N1)C(N)=O)C1=CC=CC=C1
**EK7**	0.404	CN(C)C1=C2C(CCC3=C2N=C(NC2=CC=CC(O)=C2)N=C3)=C(S1)C#N
**3TX**	0.4	OC1=CC=C(C=C1)N1C=C(N=N1)C1=NC2=CC=CC=C2C=C1
**3J7**	0.4	CC(C)(N)CNC1=C2C=CN=CC2=NC(=N1)C1=CC2=CNN=C2C=C1
**41Z**	0.394	CC1=NC2=C(C=CC=C2C(NCC2=C(C)C=CC=C2C)=C1)C(N)=O
**97K**	0.393	O=C1N=C(NC2=CC=CC=C12)C1=CC2=CNN=C2C=C1
**I0D**	0.379	CN1CCC2=C(C1)C=CC=C2NC1=C(Cl)C(=O)N(C)N=C1
**79X**	0.379	COC1=CC2=C(C=C1OC)C1=CC3=CC(O)=CC=C3N1C2=O
**4GM**	0.379	NC(=O)C1=CC=C(NCC2=CC=CC=C2O)N=C1
**IOK**	0.377	C[C@H](CCC1=CC=C(O)C=C1)NC(=O)CC1=C(NC2=CC=CC=C12)C1=CC=CC=C1
**3T9**	0.376	COC1=CC(=CC=C1O)C1=NC2=NNC(=C2C=C1)C1=CC=CC=C1
**655**	0.374	NC(=N)C1=CC=C2NC(=NC2=C1)C1=C(O)C(OC2CCCC2)=CC=C1
**WTF**	0.373	CCS(=O)(=O)C1=CC=CC=C1C(=O)N1CCN(C[C@@H]1C)C1=NC2=CC=C(F)C=C2S1
**WAM**	0.37	COC1=CC(\\C=C\\C2=[N+](C)C3=CC=CC=C3C(=C2)C(N)=O)=CC=C1O

**C70**	0.368	NC(=O)C1=C2SC(=CC2=C(N[C@H]2CCCNC2)N=N1)C1=CC=C(Cl)C=C1
**NUW**	0.367	CNC(=O)C1=CC=C2N(CC3CCN(CC3)C(C)=O)C(=NC2=C1)C1=CC(C)=C(O)C(C)=C1
**4KK**	0.365	COC1=CC=CC(CC(=O)NC2=NC(=CS2)C2=CC=NC=C2)=C1
**824**	0.365	OC1=CC2=C(NC3=C2C2=C(C(=O)NC2=O)C(=C3)C2=CC=CC=C2)C=C1
**950**	0.364	CC(C)COC1=CC=CC(C2=NC3=CC(F)=C(C=C3N2)C(N)=N)=C1O
**879**	0.364	NC1=NC(CN2C(=CC=C2C2=CC=CC=C2Cl)C2=CC=C(OC3=CN=CN=C3)C=C2)=CC=C1
**133**	0.364	CC(C)COC1=C(O)C(=CC=C1)C1=NC2=CC(F)=C(C=C2N1)C(N)=N
**G4E**	0.362	CC1=CC=CC(NC2=NNC(=N2)C2=CC=C(OC3=CC=NC=C3)C=C2)=C1C
**CJH**	0.361	CCOC1=CC=CC(=C1)C1=CC=C(NC(=O)C(C#N)C(C)=O)C=C1
**6QJ**	0.356	CC1=CC(=CC=C1O)C1=CC=CC(=N1)C(=O)C1=CC=C(F)C(O)=C1
**9ET**	0.354	CC(=O)OC[C@]1(C)OC2=C(C=C1)C1=C(C=C2C)C2=C(N1)C=C(O)C=C2
**YVQ**	0.353	N1C=C(C=N1)C1=CN2C(C=N1)=NC=C2C1=CNC2=CC=CC=C12
**Q4A**	0.352	COC1=CC2=NC(=NC(NC3CCN(CC4=CC=CC=C4)CC3)=C2C=C1OC)N1CCCN(C)CC1
**0VN**	0.35	CC(C)(C)C1=CC=C(NC2=NC3=CC(=CC=C3N2)C#N)C=C1
**QP8**	0.347	CC(C)(C)OC(=O)N1CCN(CC1)C1=CC(=NN=C1N)C1=CC=CC=C1O
**859**	0.347	NC(=O)C1=CC=CC=C1NC1=CC=NC(NC2=CC=CC(O)=C2)=N1
**6W3**	0.346	CN1C(=CC2=C1C=CS2)C(=O)NC1=CC=CC=C1COC1=CC=C(OC2CCN(C)CC2)C=C1
**C72**	0.345	NC(=O)C1=C2SC(=CC2=C(N[C@H]2CCCNC2)N=C1)C1=CC=C(Cl)C=C1

**28C**	0.345	CC1=NN2C=NN=C2C(NCCC2=CC=C(O)C=C2)=C1
**U81**	0.343	BrC1=CC2=C(OCC[C@H]2NCCCNC2=CC(=O)C3=C(N2)C=CS3)C(Br)=C1
**A3F**	0.343	COC1=CC(=CC(OC)=C1OC)C1=CC(=CN=C1N)C1=CC=CC(O)=C1
**6H2**	0.343	OC1=CC=C(C=C1O)C1=CN2C=CC=CC2=N1
**2YX**	0.341	NC1=NC(=O)C2=CC3=C(NC(NCCC4=CC=C(C=C4)C#N)=N3)C=C2N1
**ZZF**	0.34	CC1=CC=C(OC2=CC=NC(NC3=CC=C(C=C3)S(N)(=O)=O)=C2)C(C)=N1
**MCV**	0.338	COC1=CC=C(OC)C(CCC2=CSC3=NC(N)=NC(N)=C23)=C1
**8UN**	0.338	C[C@@H](C1CCCCC1)N1C2=CC=C(C=C2N=C1C1=CC2=C(OCO2)C=C1Br)C(=O)NC1=CC=C(C=C1)C#N
**PKJ**	0.337	CC1=CC=C(C=C1)C1=CSC2=NN=C(SCC(=O)NC3=CC=C4OCOC4=C3)N12
**O1Q**	0.332	CC1=CC=CC(=C1)N1N=CC=C1C1=CC(Cl)=C2N=NN(C2=C1)C1=CC2=NNC=C2C=C1
**D62**	0.332	COC1=CC=C(C=C1OC)C1=NN(C2CCN(CC2)C2=C3C=CSC3=NC(N)=N2)C(=O)[C@@H]2CC=CC[C@H]12
**CK6**	0.332	CNC1=NC(C)=C(S1)C1=CC=NC(NC2=CC=C(O)C=C2)=N1
**826**	0.332	OC1=CC=C(CN2C3=C(CCN(C3)C(=O)C3=CC=C(O)C=C3)C3=CC=CC=C23)C=C1
**KTQ**	0.331	COC1=CC=C(CCNC2=C(N=C3C=CC=CN23)C2=CC=C(C=C2)[N+]([O-])=O)C=C1
**8HZ**	0.331	CC1=CC=C(NC2=C(N=C3N2C=CC=C3C)C2=CC=C(O)C=C2)C=C1
**5ES**	0.33	OC1=CC=C(C=C1)C(=CC1=CC(NC2=CC=C(F)C=C2)=CC=C1)C1=CC=C(O)C=C1
**5C4**	0.33	CC(=C(C1=CC=C(O)C=C1)C1=CC=C(O)C=C1)C1=CC=CC(NC2=CC=CC=C2)=C1
**0N5**	0.33	COC1=CC2=C(NC3=CC=C(NC(=O)C4=CC=CC=C4)C=C3)N=CN=C2C=C1O

**70M**	0.326	OC1=C(C=C2C(CCCCN3CCN(CC3)C3=CC=C(C=C3)C#N)=CNC2=C1)C#N
**LUO**	0.325	COC1=CC=C(NC(=O)[C@H](C)NC2=NC(=O)C3=C(N2)N(N=C3)C2=CC=CC=C2C)C=C1
**K0Q**	0.325	OC1=CC=CN(CC(=O)NCCC2=CNC3=CC=CC=C23)C1=O
**9JX**	0.325	ClC1=CC=CC=C1C1=CC=CC(=C1)N1C[C@@H](CC1=O)N1CCN(CC1)C(=O)C1=NC=CS1
**4G3**	0.324	NC(=O)C1=CC=C(NCC2=CC(O)=CC=C2)N=C1
**D4Q**	0.323	NC(=O)C1=C2SC(=CC2=C(N[C@H]2CCCNC2)N=C1)C1=CC(F)=CC=C1
**RY8**	0.322	CC1=NC(=CS1)C1=CC=C(C=C1)C(=O)NC1=C(C)C(C)=CC(=C1)S(N)(=O)=O
**1DY**	0.322	COC1=CC=CC=C1NC(=O)C1=CC=C(NC(=O)CCC2=NC(=O)C3=CC=CC=C3N2)C=C1
**JGZ**	0.321	CC1=CC(=CC(C)=C1OC1=CC=NC(NC2CCN(CC2)C2=CC=C(C=C2)S(N)(=O)=O)=N1)C#N
**9HP**	0.32	OC1=CC=C(C=C1)C1=CC2=C(C=CC3=C2C=NC=C3)N=C1
**O1S**	0.319	CC1=NNC=C1C1=CC2=C(S1)C(=O)N=C(CN1CCCC1)N2
**GUK**	0.319	CC(C)NC1=CC(Cl)=NN2C(=CN=C12)C1=CC2=CNN=C2C=C1
**F1Y**	0.318	CCCC1=C(NC2=CC=C(C=C2)C(=O)NO)N2C=CC=CC2=N1
**F45**	0.317	OC1=CC=C(C=C1O)C1=NC(=CC=C1)C(=O)C1=CC=C(F)C(O)=C1
**EL2**	0.317	CNC(=O)C1=CC=C2N([C@@H]3CCC[C@@H](C3)NC(=O)C3=CC=C(Br)S3)C(=NC2=C1)C1=CC=CC=N1
**AAI**	0.317	CCCN1CCC(CC1)C1=NC2=C(C=CC=C2N1)C(N)=O
**CR3**	0.316	NC(=N)C1=CC=C2NC(=CC2=C1)C1=C(O)C(OC2CCCC2)=CC=C1
**0JA**	0.316	FC1=CC=C(OC2=CC=C3N=C(NC(=O)C4CC4)SC3=N2)C=C1NC(=O)C1=C(Cl)C(=CC=C1)C1(CC1)C#N

**3DX**	0.315	CN1CCN(CC1)C1=CC=C(C=C1)C1=CC2=C(NC3=C2C=C(N=C3)C#N)N=C1
**AI3**	0.313	COC1=CC2=CN=C3C(CC4=C3C=C3OCOC3=C4)=C2C=C1OC
**1NS**	0.313	CS(=O)(=O)NCCC1CCN(CC1)C1=C2SC(=CC2=NC=N1)C(N)=O
**Q4M**	0.312	COC1=CC=C2NC(=CC2=C1)C(=O)N1C[C@@H](CCl)C2=C3C=CNC3=CC=C12
**ALH**	0.312	CCCCC1=C(NC2=NC=CN=C12)C1=CC=C(O)C=C1
**K1H**	0.311	COC(=O)N1CCN(CC1)C1CCC(CC1)NC1=C2C=C(C=CC2=NC=N1)C#N
**E2J**	0.311	CC1=C(CCN2CCC(CC2)=C(C2=CC=C(F)C=C2)C2=CC=C(F)C=C2)C(=O)N2C=CSC2=N1
**O97**	0.31	NC(=O)C1=CC2=C(C=CC=C2S1)C1=CC=C(S1)C(=O)NC1CC1
**KTG**	0.31	OC1=CC=C(CCCNC2=CC=CC3=C2C(=O)NC3=O)C=C1
**4HN**	0.31	CCOC1=CC=CC=C1C1=CC=C(C=C1)C1=C(C#N)C(=O)C2=CN=CC=C2N1
**2WH**	0.31	CCCN1C=NC(CCNC(=O)NC2=NC3=CC=C(C=C3S2)C2=CC(OC)=CN=C2)=C1
**9Y2**	0.309	CC(C)(C)CC(=O)NC1=C(F)C=C(C=C1F)C(=O)NC1=NC=CS1
**7FW**	0.308	CN(CC1=CC2=NC=CN=C2C=C1)C(=O)C1=CC=C(C=C1)N(CC1=CC=CC=C1)C(=O)C1=CC=C(O)C=C1O
**H49**	0.307	N=C(NC1=CC=CC(CNC[C@@H]2CCCN2)=C1)C1=CC=CS1
**7IP**	0.306	COC1=CC=CC(=C1)C1=CC=CC(CCC2=CC=CC(N)=N2)=C1
**3X7**	0.306	N#CC1=CC2=C(NC3=C2C=C(C=N3)C2=CC=C(CN3CCCCC3)C=C2)C=N1
**15X**	0.305	COC1=CC=C(CC2=CC=C(NC(N)=O)C=C2)C(F)=C1C1=CC=CC(=C1)N(=O)=O
**UPX**	0.304	C\\C(=N/NC1=NC=CN1)C1=CC=C(NC(=O)C2=CC3=CC=CC(=C3N2)[N+]([O-])=O)C=C1

**LKG**	0.304	OC1=CC=C(CC2=NN=C3C=CC(=NN23)C2=CC=CC=C2)C=C1
**S79**	0.303	NC1=NC(=O)C2=CC3=C(NC(NCC4=C5C=CC=CC5=CC=C4)=N3)C=C2N1
**KA5**	0.303	COC1=CC=C(OC)C(CNC2=C(Cl)C3=C(N)N=C(N)N=C3C=C2)=C1
**5XQ**	0.303	COC1=CC=CC=C1NC1=NC(=CS1)C1=C(C)N=C(NC(C)=O)S1
**625**	0.303	NC1=NC(CN2C(=CC=C2C2=CC=CC=C2Cl)C2=CC=C(OCCCC#N)C=C2)=CC=C1
**N55**	0.302	OC1=CC=C(C=C1Cl)C1=CC=C(C=C1)[C@@H](C=C)N1C=CN=C1
**FZ9**	0.302	C1CC(=CCN1C1=NC=NC2=NNC=C12)C1=CNC2=CC=CC=C12
**2YO**	0.302	O=C1N=CNC2=CC3=C(C=C12)N=C(NCCN1CCCCC1)N3
**N58**	0.301	CN1N=C(C=C1NC(=O)NC1=CC=C(SC2=CC=NC=C2)C=C1)C(C)(C)C
**EYF**	0.301	COC1=CC=CC(CN2C=CC3=CC(=CN=C23)C(=O)NO)=C1
**2YM**	0.301	NC1=NC(=O)C2=CC3=C(NC(NCCN4CCCCC4)=N3)C=C2N1
**4L7**	0.3	BrC1=CC=C(S1)C(=O)[C@H]1CNC[C@@H]1C1=CC=C2C(=O)N=CNC2=C1
**926**	0.3	CN1C=CN=C1SC1=CC=CC(=C1)C1=CC=CC(=O)N1
**Y6V**	0.299	O=C(N(CC1=CC=CS1)C1CC1)C1=CC=C2NC(=O)COC2=C1
**IEO**	0.299	NC(=O)C1=C2NC=C(C#N)C2=C(OC2CCNCC2)C=C1
**HZY**	0.299	COC1=CC(C[N+]2=CC=C3C=C(OS(N)(=O)=O)C=CC3=C2)=CC(OC)=C1OC
**H2K**	0.297	ClC1=CC(NC(=O)NC2=CN=C(C=N2)C#N)=C(O[C@@H]2CCNC2)C=C1
**8E6**	0.297	NC(=O)C1=C2N=C(NC2=CC(F)=C1)C1=CC2=C(CCNC2)S1

**37R**	0.297	NC(=O)CCCCC1=C(O)C=C2OC3=C(NC2=C1)C=CC(O)=C3
**2YV**	0.297	NC1=NC(=O)C2=CC3=C(NC(NCCC4=CC=CC=C4)=N3)C=C2N1
**1RQ**	0.296	OC1=CC=C2N=C(N(C2=C1)C1=CC=NC(N[C@@H]2CCCN(C2)C(=O)C2CC2)=N1)C1=CC2=CC=CC=C2C=C1
**07Q**	0.295	COC1=CC(=CC=C1)[C@@H](C)NC(=O)NC1=NC(=CS1)C1=CC=NC=C1
**ZZW**	0.294	ONC(=O)C1=CC2=C(C=N1)N(CC1=CC=C(F)C=C1)C1=C2C=CC=C1
**DLN**	0.294	CC(C)N1N=C(C#CC2=CC=CC(O)=C2)C2=C(N)N=CN=C12
**9N2**	0.294	OC1=CC(=CC=C1F)C(=O)C1=NC2=CC(=CC=C2C=C1)C#N
**3KC**	0.294	OC1=CC=C(C(Cl)=C1)C1=NC2=C(C=CC=C2)C2=CC=NC3=C2C1=CN3
**N61**	0.293	CN1N=C(C=C1NC(=O)NC1=CC=C(OC2=CC=NC=C2)C=C1)C(C)(C)C
**EWV**	0.293	COC1=CC2=C(C=C1OC)C1=CC3=CC=C4OCOC4=C3C=[N+]1CC2
**770**	0.293	ClC1=CC=CC=C1C1=CC2=C(C3=C(N2)C=CC(NC=O)=C3)C2=C1C(=O)NC2=O
**QK0**	0.292	CC(=O)NC1=NC2=CC=C(C=C2S1)C1=CC(NS(=O)(=O)C2=CC=C(F)C=C2)=C(Cl)N=C1
**8TK**	0.292	C[C@H](NC1=C(C#N)C(N)=NC=N1)C1=C(C(=O)N2C(C)=CSC2=C1)C1=CC=CC=C1
**0WP**	0.292	COC1=C2C=C(NC(=O)C3=C(F)C(NC(=O)C4=CC(=CC=C4)C(C)(C)C#N)=CC=C3F)C=NC2=NN1
**P8Z**	0.291	CN1CCN(CC1)C1=CC=CC=C1CNC1=C(C)C(=O)N(C)N=C1
**JVT**	0.291	CN1CCN(CC1)C1=CC=C(NC(=O)C2=NNC3=CC=CC=C23)C=C1
**DTM**	0.291	COC1=CC(CN(C)C2=CC3=C(N)N=C(N)N=C3N=C2)=CC(OC)=C1OC
**9MK**	0.291	CC1=CC2=CC=C(N=C2C=C1)C(=O)C1=CC=C(F)C(O)=C1

**8PV**	0.291	O=C(NCC1=CC=CC=C1)C1=CC=CC(NCC2=NN=C(N2)C2=CC=NC=C2)=C1
**VXS**	0.289	OC1=CC=CC=C1CCCNC1=CC=CC2=C1C(=O)NC2=O
**MEY**	0.289	OC1=CC(O)=C(Cl)C=C1N1C(=O)NC2=CC(CNS(=O)(=O)C3=C4C=CC=CC4=CC=C3)=CC=C12
**6GE**	0.289	CC1=CC=C(O)C=C1NC1=CC=NC2=CC=C(C=C12)S(C)(=O)=O
**5DG**	0.289	CC(=C(C1=CC=C(O)C=C1)C1=CC=C(O)C=C1)C1=CC=CC(NC2=CC=C(F)C=C2)=C1
**N9W**	0.288	CN1CCC2=C(C1)C1=CC=CC=C1N2CC1=CC=C(C=C1)C(=O)NO
**JCW**	0.288	NC(=O)C1=CC(=NC=C1)C1=CC(=CN=C1F)[C@H]1C[C@@H]2CC[C@H]1N2
**7DZ**	0.288	CC(C)CCN1[C@@H](C)C(=O)N(C)C2=CN=C(NC3=CC(F)=C(O)C(F)=C3)N=C12
**FJU**	0.287	CCC1=CC(NC(=O)C2=CC3=CC=CC(O)=C3OC2=N)=CC=C1
**64N**	0.287	CCC(=O)NC1=CC(Cl)=C(OC2=CC=C(O)C(=C2)C2=CC=C(C=C2)C(N)=O)C(Cl)=C1
**MDY**	0.286	COC1=CC=CC=C1C1=CC=C(NC(=O)C(C#N)C(C)=O)C(Cl)=C1
**5GD**	0.286	COC1=CC(OC)=C2C(=O)N=C(NC2=C1)C1=CC(C)=C(OCCN2CCCC2)C(C)=C1
**58W**	0.285	OC1=CC(O)=C(Cl)C=C1NC(=O)C1(CCC1)C1=CC=CC=C1
**2KL**	0.285	CCNC(=O)C1=CC2=C(N=C(N)N=C2S1)C1=CC(OCCN2CCCC2)=C(Cl)C=C1Cl
**LXX**	0.284	N1C=C2C=C(C=NC2=N1)C1=CC2=C(C=CN=C2C=C1)C1=CC=NC=C1
**L26**	0.284	CCC1=C(NC(C)=C1C(C)=O)C(=O)NC1=CC=CC(OCC(N)=O)=C1
**5NW**	0.284	COC1=CC(=CC=C1NC1=NC2=C(NCC(C)(C)C)N=CC=C2C=N1)C1=CN(C)N=C1
**4Q3**	0.284	CC(C)N1N=C(NC(C)=O)C=C1C1=CC=C(N(C)C(=O)C2=C(F)C=CC=C2Cl)C(=C1)N1C[C@@H]2C[C@@H]2C1

**17V**	0.284	CC(C)N1N=CN=C1C1=CC2=C(S1)C1=CC=C(C=C1OCC2)C(N)=O
**75X**	0.283	CC1=C(C=CC=C1O)C1=NC2=NC(N)=NC(OCC3CCCCC3)=C2N1
**GQW**	0.282	ONC(=O)C1=CC=C(Cl)C(NC(=O)C2=C3SC=CC3=CC=C2)=C1
**4H7**	0.282	CN1CCN(CC1)C1=CC=CC=C1CNC1=CC=C(C=N1)C(N)=O
**GXK**	0.281	ClC1=NN2C(=CN=C2C(NCC2CC2)=C1)C1=CC2=CNN=C2C=C1
**G6J**	0.281	CCCN1[C@H](C)C(=O)N(CCC)C2=CN=C(NC3=CC(F)=C(O)C(F)=C3)N=C12
**0CV**	0.281	COC1=CC2=C(N3CCNCC3)C3=C(C)NN=C3N=C2C(C)=C1
**J2V**	0.28	COC1=CC=C(C=N1)C1=C(NC2=CC=CN=C12)C1=CC=NC(NC(C)=O)=C1
**G6T**	0.28	NC1=NC(OCC2CCCCC2)=C2NC(=NC2=N1)C1=CC(=CC=C1)S(N)(=O)=O
**5CC**	0.279	CCC(=C(C1=CC=C(O)C=C1)C1=CC=C(O)C=C1)C1=CC=CC(NC2=CC=CC=C2)=C1
**43A**	0.278	COC1=CC(=CC=C1O)C1=CC=C2C(NC(=O)C2=CC2=CC=CN2)=C1
**41E**	0.278	COC1=CC(NC(=O)CCC2CCN(CC2)C(=O)C2=CC=C(S2)C(C)=O)=CC=C1
**SV8**	0.277	O=C1NCC2=C1NC=C2C1=CN=C2NC=C(CC3=CC=CC=C3)C2=C1
**DUL**	0.277	CCSC1=CC=CC(=C1)C1=CC=C(S1)C(N)=O
**V5G**	0.276	NC1=NC(CCC2=CC(OCC3=CC=C4C=CC(N)=NC4=C3)=CC=C2)=CC=C1
**HVU**	0.276	CN1CCC(CC1)C1=CNC2=CC=C(O)C=C12
**4P9**	0.276	OC1=CC=C(\\C=N\\NC2=CC=C(Cl)C=C2)C(O)=C1O
**43H**	0.276	OB(O)C1=CN(C=C(O)C1=O)C1=CC=CC(=C1)C1=CC=CC=C1

**8C1**	0.275	CC(C)OC1=CC2=C(O[C@@H]3CCCNC3)C=CN=C2C=C1C(N)=O
**43R**	0.275	COC1=CN=CC(=C1)N1CCC2=C(C1)SC(NC(C)=O)=N2
**0NV**	0.275	C(N1CCC(CC1)N1C=NC2=C1C1=C(NC=C1)N=C2)C1=CC=CC=C1
**99S**	0.274	COC(=O)C1=CC=C(S1)C1=CC(=CC(=C1)C#N)[C@]1(C(C)C)C2=C(C)NN=C2OC(N)=C1C#N
**584**	0.274	CC(C)CCN1[C@H](C)C(=O)N(C)C2=CN=C(NC3=CC(F)=C(O)C(F)=C3)N=C12
**KT4**	0.273	OC1=CC=C(CCCNC2=CC=CC3=C2C(=O)NC3=O)C(O)=C1
**GQB**	0.273	Nc1[nH]c2ccc(CNC(=O)C3=CC4=CC=CC=C4N3)cc2s1
**E5M**	0.273	C[C@@H]1N(C)C2=NC(NC3=CC(F)=C(O)C(F)=C3)=NC=C2N(C)C1=O
**COQ**	0.273	COC1=CC(OC)=CC(CN(C)C2=CC3=C(N)N=C(N)N=C3N=C2)=C1
**NVV**	0.272	IC1=CC=CC(=C1)C(=O)[C@H]1CNC[C@@H]1C1=CC=C2C(=O)N=CNC2=C1
**R8E**	0.271	NC1=NC2=NNC(COC3=CC(OC4=CC(=CC(Cl)=C4)C#N)=C(Cl)C=C3)=C2C=C1
**JZH**	0.271	OC1=CC=C(C=C1)[C@H]1C2=CNC3=NC=CC(=C23)C2=CC=CC=C2NC1=O
**8Q7**	0.271	COC1=CC=C(CN2C3=C(C4=C(CN(CC4)C(=O)[C@@H]4CC4(F)F)S3)C3=NC=NN3C2=O)C=C1
**5JN**	0.271	CC1=CC(NC(=O)CC2=CC=C(OC(C)(C)C(=O)OCCO[N+]([O-])=O)C=C2)=CC(C)=C1
**4FX**	0.271	COC1=CC=CC=C1CNC1=CC=C(C=N1)C(N)=O
**NUM**	0.27	OC1=CC=C(CCNC(=O)C2=CC=CC=N2)C=C1
**E78**	0.27	C[C@@H](C1CC1)N1CC2=C(C1=O)C(C)=CC(=C2)C1=C(C)N=C(NC(C)=O)S1
**35O**	0.27	COC1=CC=C(NC(=O)[C@@H](C)NC2=NC(=O)C3=C(N2)N(N=C3)C2CCCC2)C=C1

**JEW**	0.268	NC1=NNC(=C1)C1=CC=C2C=CN(CC3=CC=CC=C3C#N)C2=C1
**5O1**	0.267	COC1=CC(=CC=C1NC1=NC2=C(NC3CCCCC3)N=CC=C2C=N1)C1=CN(C)N=C1
**3ND**	0.267	ClC1=CC=C(C=C1)[C@@H]1CNC[C@H]1C(=O)NC1=C(Cl)C=C2C(=O)NC=CC2=C1
**3DC**	0.267	N(C1=CC=NC=C1)C1=NC=NN2C=C(C=C12)C1=CNN=C1
**30J**	0.267	C[C@@H]1CN(CCN1C1=CC=C(C=N1)C#N)C1=C2C=C(Cl)C=CC2=C(CC2=CC=CC=C2)N=N1
**16L**	0.267	CC1=CC(\\C=C\\C2=CC=CC(NC(=O)C=C)=C2)=CC(C)=C1O
**9F4**	0.266	ONC(=O)[C@@H]1[C@@H]([C@H]1C1=CC=C(C=C1)C1=NC=C(F)C=N1)C1=CC=CC=C1
**3XK**	0.266	CC(=O)NC1=CC=CC(=C1)C1=CC2=C(NC3=C2C=C(N=C3)C#N)N=C1
**S1W**	0.265	CN(C)C1=CC=CC(OC2=CN=C(NC3=CC=CC(O)=C3)N=C2)=C1
**CO4**	0.265	COC1=CC(=CC(OC)=C1OC)N(C)CC1=C(C)C2=C(N)N=C(N)N=C2N=C1
**666**	0.265	C[C@@H]1CC(=O)NN=C1C1=CC=C(NC2=C(CC3=CC=CC(I)=C3)C(=O)CCC2)C=C1
**ZSO**	0.264	FC1=CC=C(C=C1)[C@@H]1CCNC[C@H]1COC1=CC2=CNN=C2C=C1
**LTQ**	0.264	CC1=CC(=CC=C1N1CCN(CC1)S(=O)(=O)N1CSC[C@@H]1C(=O)NO)C#N
**9ST**	0.264	CN1C[C@@H](C[C@@H](C1)C1=CC=CC=C1)NC1=C(Br)C(=O)N(C)N=C1
**5B2**	0.264	NC1=NNC2=CC=C(C=C12)C1=CC=CC=C1
**15F**	0.264	ClC(Cl)(Cl)C(=N)OCC1=CC=[N+](CC2=CC=CC=C2)C=C1
**YDJ**	0.263	NC(=O)NC1=C(SC(=C1)C1=CC(F)=CC=C1)C(=O)N[C@H]1CCCNC1
**RKZ**	0.263	C[C@H](CN1C(C)=NC2=CC=C(N=C12)C1=CC(N)=NC(N)=C1)OC1=CC=CC=C1

**6JU**	0.263	ONC(=O)CN1C=C(CC2=CC=CC=C2)C2=CC(Br)=CC=C12
**5H2**	0.263	COC(=O)NC1=CC=C2C3=C(CCOC2=C1)C=C(S3)C1=NN=CN1C1=CC=CC=C1Cl
**3FR**	0.263	C[C@H](NC1=NC=C2C(C)=NN(C2=C1)C1=CC(=CC(Cl)=C1)C1=CC=CC=C1C(N)=O)C1=CC=CC=C1
**304**	0.263	CC[C@@H](C)NC1=NC=C(S1)C(=O)NC1=CC(=CC=C1C)C(=O)NC
**92Q**	0.262	CC1=CC=CC(Cl)=C1NC(=O)C1=CN=C(NC2=CC=CC(=C2)C(=O)N[C@H]2CCNC2)S1
**ZVZ**	0.261	CNC(=O)COC1=CC=C(C(CN2CCN(CC2)C2=CC=CC=N2)=C1)C1=CC=C(C=C1)C#N
**BER**	0.261	COC1=C(OC)C2=C[N+]3=C(C=C2C=C1)C1=C(CC3)C=C2OCOC2=C1
**3GN**	0.261	C[C@]1(CCCN1)C1=NC2=C(C=CC=C2N1)C(N)=O
**S8B**	0.26	CC(C)NC(=O)C1=CC=C(C=N1)C#CC1=CC=NC=C1
**RT3**	0.26	CC1=CC=C2N(CCCC2=C1)C(=O)SCC(=O)NC1=CC=C(C=C1Cl)S(N)(=O)=O
**GQ5**	0.26	C[C@H]1CCCN1CC1=NC2=CC(NC(=O)C3=CC=C4N(C)N=CC4=C3)=CC=C2N1
**3L0**	0.26	C[C@H]1N2C(COC3=CC(C4=CC=CC=C4)=C(C=C23)N(C)C2(C)CN(C)C2)=NNC1=O
**3E1**	0.26	CC(=O)NC1=CC=C(O)C(=C1)C1=CC(NC(C)=O)=CC=C1O
**18A**	0.26	CCC(=O)NC1=CC=C(\\C=C\\C2=CC(C)=C(O)C(C)=C2)C=C1
**M94**	0.258	CNCCC1=CC=CC(OCC2=CC3=NC(N)=CC=C3C=C2)=C1
**5B4**	0.258	CN1C=C(C=N1)C1=CC(OC2=CC(F)=C(NC(=O)C3(CC3)C(=O)NC3=CC=C(F)C=C3)C=C2F)=CC=N1
**1NR**	0.258	CC(C)(C)C(=O)NCCC1CCN(CC1)C1=C2SC(=CC2=NC=N1)C(N)=O
**A27**	0.257	CCCC(=O)NC1=NNC2=CC(C3=CC=C(O)C=C3)=C(C=C12)C1=CC=CC=C1

**BAI**	0.256	NC(=N)C1=CC=C2NC(CC3=NC4=CC=CC=C4N3)=NC2=C1
**5H5**	0.256	CC(C)N1N=CN=C1C1=CN2CCOC3=CC(O[C@@H](C)C(N)=O)=CC=C3C2=N1
**LKQ**	0.255	COC1=NN(C)C=C1NC1=NC=C(C)C(=N1)C1=CNC2=C(NC(=O)C3=CC=CN=C3C)C=CC=C12
**7DQ**	0.255	CC(C)C1=CC=C(CC(=O)N2CCN(CC2)C2=CC=C(N=N2)C#N)C=C1
**R69**	0.254	CN(C)C1=CC=C(O)C(=C1)C(=O)C1=CC=C(C=C1)C(=O)N[C@@H]1CCCNC[C@H]1NC(=O)C1=CC=NC=C1
**JA1**	0.254	CCCCCCC1=CC=C(OC2=CC=C(C=C2)[N+]([O-])=O)C(O)=C1
**EMW**	0.254	COC1=CC=C(NC(=O)N2CCN([C@@H](C)C2)C2=C3C=NN(C)C3=NC(N)=N2)C(C)=C1
**ABZ**	0.254	NC1=NC(NC2=CC=C(C=C2)C#N)=NC(CC2=C3C=CNC3=CC=C2Cl)=N1
**9LP**	0.254	OS(=O)(=O)OC1CCC(CC1)NC(=O)[C@H](C1CCCCC1)N1C2=CC(F)=C(F)C=C2N=C1C1=CC=C(Cl)C=C1
**KT1**	0.253	COC1=CC(O)=CC=C1CCCNC1=CC=CC2=C1C(=O)NC2=O
**68M**	0.253	C[C@H](CC1=CC2=CC(=CC=C2N=C1N)C1=NC=CC=C1C)C(=O)NCCC(C)(C)C
**58G**	0.253	NC(=O)C1=CC=C(N[C@@H]2CC[C@@H](C2)C2=CC=CC=C2)N=C1
**50B**	0.253	COC1=C(OC)C2=C[N+]3=C(C(CCCC(C4=CC=CC=C4)C4=CC=CC=C4)=C2C=C1)C1=CC2=C(OCO2)C=C1CC<br>3
**SK6**	0.252	CC1=CC(CCC2=CC(=CC(NCCC3=CC=CC=N3)=C2)C#N)=NC(N)=C1
**4G4**	0.252	CN(C)C1=CC=CC=C1CNC1=CC=C(C=N1)C(N)=O
**Z7H**	0.251	COC1=CC=C(OC2=C(Cl)C=C(NC(=O)CC3=CC=CN=C3)C=C2Cl)C=C1C(C)C
**U1T**	0.251	CC1=C2C=C(NC(=O)C2=CC=C1)C1=CC=C(CN2CCCC2)C=C1
**MXI**	0.251	COC1=CC2=CC=C(C=C2C=C1)C1=C(NC(=N1)C(C)(C)C)C1=CC=NC(N)=C1

**CTI**	0.251	COC1=C(OC)C2=C(C=C1)C1=C(C3=C(C=C1)C=C1OCOC1=C3)[N+](C)=C2
**78P**	0.251	C[C@@]1(CCCN1)C1=NC2=C(C=CC=C2N1)C(N)=O
**3FV**	0.251	ClC1=CN(N=C1C(=O)NC1=CNN=C1C1=CC=CC=N1)C1CCNCC1
**WXV**	0.25	CNC(=O)C1=NN(C)C2=C1C=CC1=C2N=C(NC2CCN(CC2)S(C)(=O)=O)N=C1
**NVS**	0.25	COC1=CC=C(C=C1)N1CC2=CC=C(O)C=C2OC1=O
**EX7**	0.25	OC1=C2N=C(CCC3=CC(Cl)=CC=C3)NC2=CC=C1
**43G**	0.25	OC1=CN(C=CC1=O)C1=CC(=CC=C1)C1=CC=CC=C1
**JC8**	0.249	NC(=O)C1=CN=C(C=C1)C1=CC(=CN=C1F)[C@H]1C[C@@H]2CC[C@H]1N2
**E89**	0.249	COC1=CC=C(CCC2=C3NC=NC3=CC3=C2NC(N)=NC3=O)C=C1
**C51**	0.249	C[C@H](C1CCC(CC1)C1=CC=NC2=CC=CC=C12)C(=O)NC1=CC=C(C=C1)C#N
**RKK**	0.248	CCCCN1C(CCCC)=NC2=CC=C(N=C12)C1=CC(N)=NC(N)=C1
**9ME**	0.248	OC1=CC(=CC=C1F)C(=O)C1=NC2=CC=CC=C2C=C1
**6GH**	0.248	NC1=NC(=CC(=N1)C1=CSC=C1)N(CC1=CC=CC=N1)CC1=CC=CC=N1
**924**	0.248	NC1=C2C(SC=C2C2=CC3=C(C=C2)N(CC3)C(=O)CC2=CC=CC=C2)=CC=N1
**H5G**	0.247	OC1=CC=CC(CC(=O)NC2=CC=CC(Cl)=C2)=C1
**52D**	0.247	O=C(C1=CC=NC=C1)C1=CC=C(OCCN2CCCC2)C=C1
**19Z**	0.247	NC(=O)C1=CC(=CC=C1NCC1=CC=C(O)C=C1)[N+]([O-])=O
**QV5**	0.246	CC(C)OC1=CC(=CC=C1C1=C(NC2=CC(F)=C(Cl)C=C12)C(N)=O)N1CCNCC1

**H0M**	0.246	OC1=CC(CC(=O)NC2=CC=CC=N2)=CC=C1
**D90**	0.246	CN1CCC2=C(C1)SC(=N2)C(=O)NC1=CC=CC=C1NC(=O)C1=CC2=CC(Cl)=CC=C2N1
**6WW**	0.246	CN([C@H]1CC[C@H]2[C@@H]3CCC4=CC(O)=CC=C4[C@H]3CC[C@]12C)C1=CC=CC=C1
**6BU**	0.246	O=C(NC1=NNC2=CC(=CC=C12)C1=CC(NS(=O)(=O)C2CC2)=CC=C1)C1CC1
**2HV**	0.246	CC(=O)NC1=NC(=CS1)C(=O)NC1=CC=CC=C1N1CCCCC1
**9HJ**	0.244	CCCN(C)C1=NC2=CC(OC)=C(OC)C=C2C(NC2CCN(C)CC2)=N1
**8ZZ**	0.244	CC1=CC=CC(Cl)=C1NC(=O)C1=CN=C(NC2=CC=CC(=C2)C(=O)N[C@@H]2CCNC[C@H]2F)S1
**8ZH**	0.244	CC1=CC=CC(Cl)=C1NC(=O)C1=CN=C(NC2=CC=CC(=C2)C(=O)NC2CCNCC2)S1
**65O**	0.244	CC1=C(SC2=CC=CC3=CC=CC=C23)SC2=NC(N)=NC(N)=C12
**2UQ**	0.244	N=CC1=NC=C(C=N1)C(=O)NCC1=CC=CC=C1
**VXV**	0.243	OC1=CC(Cl)=CC(CCCNC2=CC=CC3=C2C(=O)NC3=O)=C1
**FAZ**	0.243	CN(C(=O)C1=CC2=C(S1)C1=CC=C(NC(C)=O)C=C1OCC2)C1=CC=CC=C1Cl
**C4Q**	0.243	C(N1CCNCCNCCNCC1)C1=C2C=CC=CC2=NC=C1
**45R**	0.243	COC1=CC(=CC=C1C(=O)NC1=CC=C2CCNCCC2=C1)C1=CNN=C1
**Z68**	0.242	NC1=C(SC(NC2CCCCC2)=N1)C(=O)C1=CC(=CC=C1)[N+]([O-])=O
**QPC**	0.242	CC(C)(C)CNC(=O)CC1=CC=C(NC2=C(C=NC(=N2)C2=CC=CC=C2)C(N)=O)C=C1
**JHT**	0.242	CN(C)C1=CC=CC(CNCC2=CC3=NC(N)=CC=C3C=C2)=C1
**9P1**	0.242	CN(C)C1=CC=C(CNCC2=CC3=NC(N)=CC=C3C=C2)C=C1

**76B**	0.242	CC1=CC(=CC=N1)C1=NN=C(CCNC(=O)C2=CC(=CC(O)=C2O)C2=CC=C(F)C=C2)N1
**7U6**	0.241	NC(=O)C1=C2N=C(NC2=CC=C1)C(=O)N1CCNCC1
**5UO**	0.241	ClC1=CC=C(C=C1)C1CCN(CCC2=CN(N=C2)C2=C3NC=NC(=O)C3=CC=N2)CC1
**516**	0.241	CN[C@@H](C)C(=O)N[C@@H](C1CCCCC1)C(=O)N1CCC[C@H]1C1=NC(=CS1)C1=CC=CC2=CC=CC=C12
**W4G**	0.24	O=C1N(CC2=CC=CC=C2)C=NC2=CC(=CC=C12)C1=CNN=C1
**T61**	0.24	ONC(=O)C1=CC=C(Cl)C(NC(=O)C2=CC=CC=C2)=C1
**IDK**	0.24	O=C(NC1=NNC2=CC(=CC=C12)C1=CC(NS(=O)(=O)CC2CC2)=CC=C1)C1CC1
**8CG**	0.24	CC[C@H]1[C@@H](COC2=C3C=C(OC)C(=CC3=CC=N2)C(N)=O)NC(=O)[C@H]1F
**RFM**	0.239	O=C(CN1N=NC2=CC=CC=C12)N(CC1=CSC=C1)C1=CC=C(NC(=O)C2=CC=CC=C2)C=C1
**O1H**	0.239	ClC1=C2N=NN(C2=CC(=C1)C1=CC=NN1CC1=CC=C(C=C1)C#N)C1=CC2=NNC=C2C=C1
**KZQ**	0.239	CC1=C([C@@H](NC(=O)N1)C1=CC=C(F)C=C1)C(=O)NC1=CC2=CNN=C2C=C1
**426**	0.239	NC(=N)C1=CC2=CC=C(C=C2C=C1)C(=O)NC1=CC=CC(OC2CCCC2)=C1
**VUG**	0.238	CC1=CC=CC(=C1)C1CCC(CC1)N1CCN(CC1)C1=CC=CN=C1
**4G0**	0.238	CN(C)C1=CC=CC(CNC2=CC=C(C=N2)C(N)=O)=C1
**VXM**	0.237	OC1=CC=C(F)C=C1CNC1=CC=CC2=C1C(=O)NC2=O
**QWI**	0.237	C1C[C@H](CC2(CCN(CC2)C2=C3NC=NC3=NC=N2)N1)C1=CC=CC=C1
**IXF**	0.237	CC(C)C1=CC=C(C=C1)N(C)C1=CC2=C(N)N=C(N)N=C2N=C1
**7O3**	0.237	OC1=CC=C(Cl)C(NC2=C3C=CC(NC(=O)CCl)=CC3=NC=N2)=C1

**TWP**	0.236	ONC(=O)C1=CC=C(CN2C=CC3=CC=CC=C23)C=C1
**PRD**	0.236	COC1=CC=C(OC)C(CN(C)C2=CC3=C(N)N=C(N)N=C3N=C2)=C1
**DEH**	0.236	COC1=CC=C2C=C3N(CCC4=C3C=C(O)C(OC)=C4)C=C2C1=[OH+]
**8QE**	0.236	CC(C)(C)NC1=C(C#N)C(N)=CC(NC2=CC=C(C=C2)C(N)=O)=N1
**TMU**	0.235	COC1=CC=C(CNC(=O)NC2=NC=C(S2)[N+]([O-])=O)C=C1
**M8G**	0.235	ONC(=O)\\C=C\\C1=CC=CC=C1NC(=O)C1=CC=CC=C1OC1=CC=CC=C1
**K8M**	0.235	FC1=CC(=CC(=C1)C1=CC=NC=C1)C(=O)N1C[C@@H](CC(F)(F)C1)C(=O)NC1=CC=C(Cl)C=C1
**5UL**	0.235	ClC1=CC=C(CC2CCN(CCC3=CN(N=C3)C3=C4NC=NC(=O)C4=CC=N3)CC2)C=C1
**XU2**	0.234	O=C(NC1=CC=C(NC2=CC=NC3=C2C2=C(C=CC=C2)C(=O)N3)C=C1)C1=CC=CC=C1
**X5M**	0.234	CCN(CC)C1=CC=C(C=C1[N](O)=O)C1=NNC(=O)C[C@H]1C
**WDY**	0.234	O=C(NC1=CC=CC(=C1)C1=NNC=C1)C1=CC=CC2=CNN=C12
**TJH**	0.234	OC1=CC=CC2=C1C(=O)C=C(NCCNC1=C3C=CC(Cl)=CC3=NC3=C1CCCC3)C2=O
**HJM**	0.234	CC[C@H]1C[C@@H]2C[C@@H]3[C@H]1N(C2)CCC1=C3NC2=CC=C(OC)C=C12
**5Q2**	0.234	CN1CCN(CC1)C1=CC=C(NC(=O)C2=C(NCCC3=CC=C(Cl)C=C3)C=CNC2=O)C=C1
**16X**	0.234	CC1=C(SC(N)=N1)C1=CC=NC(NC2=CC=CC(=C2)N2OO2)=N1
**0P8**	0.234	COC1=C(CNCCCNC2=CC(=O)C3=CC=CC=C3N2)C=C(Br)C=C1Br
**S9H**	0.233	COC1=CC=C(NC2=NC=CC(=N2)C2=CC=C3NC(=O)CCCC3=C2)C=C1
**279**	0.233	CCNC(=O)C1=CN2N=CN=C(NC3=CC(=CC=C3C)C(=O)NOC)C2=C1C

**Q26**	0.232	CCOC1=CC=C(C=C1C1=CC2=NC(N)=NC(N)=C2C=C1)C1=CC=CC(NS(C)(=O)=O)=C1
**KSY**	0.232	OC1=CC=CC(CCCNC2=CC=CC3=C2C(=O)NC3=O)=C1
**E1D**	0.232	C[C@H]1N(C)C2=NC(NC3=CC(F)=C(O)C(F)=C3)=NC=C2N(C)C1=O
**9S1**	0.232	NC1=CC=C2NC(=CC2=N1)C1C=NN(C1=O)C1=CC(CNC(=O)NC2=CC=CC=C2)=CC=C1
**QRX**	0.231	CCCN1C(CNC2=CC=CC(=C2)C(=O)NCC2=C(F)C=CC=C2F)=NN=C1C1=CC=NC=N1
**LKT**	0.231	ClC1=CC=CC(N2CC[C@@H](C2)[C@H](CC#N)N2C=C(C=N2)C2=C3C=CNC3=NC=N2)=C1C#N
**LD2**	0.231	O=C1COC2=CC=C(C=C2N1)C(\\CC1=CC=CC=C1)=N\\N1CN=NC1=S
**K4A**	0.231	CCN1N=CC(=C1C)C1=NC2=C(N=CN=C2N1C)N1CCC(CC1)N1C(=O)NC2=NC=CC=C12
**KTM**	0.23	OC1=CC=CC=C1CNC1=CC=CC2=C1C(=O)NC2=O
**GVK**	0.23	ClC1=CC=C(C=C1)C1(CCNCC1)C1=CC=C(C=C1)C1=C2N=CNC2=NC=N1
**FH3**	0.23	CN1C=C(C(C)=N1)C1=NC2=NC=C(Cl)C(N3CC[C@@H](CNC(C)=O)C3)=C2N1
**7KZ**	0.23	CCCCCCNC1=NC2=CC(OC)=C(OC)C=C2C(NC2CCN(C)CC2)=N1
**65C**	0.23	CC1=CNC(=N1)C1=CN=C(NCCNC2=CC=C(C=N2)C#N)N=C1C1=CC=C(Cl)C=C1Cl
**4YU**	0.23	COC(=O)C=CCNC(=O)C1=C(C)N(C)C2=CC=C(OC)C=C12
**W45**	0.229	SCC(=O)NCCCCCN1C=CC2=CC(Cl)=C(Cl)C=C12
**328**	0.228	COC1=CC=NC(NC2CCN(CC2)C(=O)C2=CC=C(C=C2)C#N)=C1
**SM6**	0.227	CCOC(=O)C1=C(NC2=CC3=CCC(NO)=C3C=C2)C2=CC=NC=C2S1
**JCE**	0.227	NC1=NNC(=C1)C1=CC=C2C=CN(CC3=CC=CC=N3)C2=C1

**4TV**	0.227	CN1C=C(C=N1)C1=CC=C(C=C1)C1=CN=CC(Cl)=C1N1CCC2(CCNC2=O)CC1
**T27**	0.226	CC1=CC(\\C=C\\C#N)=CC(C)=C1NC1=CC=NC(NC2=CC=C(C=C2)C#N)=N1
**JDQ**	0.226	NC1=NNC(=C1)C1=CC=C2C=CN(CC3=CC=CC=C3)C2=C1
**81F**	0.226	CC1=CC=CC(=C1)C1CCC(CC1)N1CCN(CC1)C1=CC(Br)=CN=C1
**5TD**	0.226	O=C(CCC1CCN(CC1)C1=CC=C(C=C1)C#N)N1CCCC1
**56Y**	0.226	CCC1=C(NC(C)=C1C(C)=O)C(=O)N(C)CC1=CC=CC(=C1)C(N)=O
**C9J**	0.225	CN1C=CC2=CC=C(N=C2C1=O)C1=CN(N=N1)C1=CC=C(O)C(F)=C1
**6WV**	0.225	C[C@]12CC[C@H]3[C@@H](CCC4=CC(O)=CC=C34)[C@@H]1CC[C@@H]2NC1=CC=CC=C1
**YKC**	0.224	O=C(N[C@@H]1C2=CC=CC=C2C2=C(C=CC=C12)C1=NC2=CC=NC=C2N1)C1=CC=NC=C1
**VM4**	0.224	C[C@@H](SC1=NC2=C(C=NN2C)C(=O)N1C1=CC=CC=C1)C1=CC=CC(O)=C1
**L74**	0.224	ClC1=CC=C(C=C1)N1CCN(CC2=CNC3=NC=CC=C23)CC1
**H6K**	0.224	O=C(NC1=NNC(=C1)C1=CC=CC=C1)NC1=CN=C(C=N1)C#N
**EKT**	0.224	CC(C)CCN1C2=C(C=NC(NC3=CC(F)=C(O)C(F)=C3)=N2)N(C)C(=O)C1(C)C
**EDH**	0.224	CCCN1C(NC(=O)C2=CC=CC(=C2)C(N)=O)=NC2=CC=CC=C12
**C73**	0.224	NC(=O)NC1=C(C=C(S1)C1=CC=C(Cl)C=C1)C(=O)N[C@H]1CCCNC1
**WR2**	0.223	CNC(=O)[C@@H](NC(=O)[C@H](CC(C)C)[C@H](CNC(=O)C1=NC=CS1)C(=O)NO)C(C)(C)C
**RK8**	0.223	C[C@@H](CN1C(C)=NC2=CC=C(N=C12)C1=CC(N)=NC(N)=C1)OC1=CC=CC(Br)=N1
**QKQ**	0.223	COC1=CC=C(C=C1)C1=CN=CC2=CC=C(N=C12)C(=O)NCCCN1C=CN=C1

**7Z0**	0.223	COC1=NNC2=NC=C(C=C12)C#CC1=C(F)C=CC(NS(=O)(=O)C2=CC=CC(Br)=C2)=C1F
**64O**	0.223	CCC(=O)NC1=CC(Cl)=C(OC2=CC=C(O)C(=C2)C2=CC=C(NC(N)=O)C=C2)C(Cl)=C1
**172**	0.223	NC1=NC2=CC(O)=CC=C2N1
**7AH**	0.222	FC1=CC=C(CC(=O)NC2=CC=C3NC=C(C4CCNCC4)C3=C2)C=C1
**6GM**	0.222	NC1=NC(=CC(=N1)C1=CC=C(O)C=C1)N(CC1=CC=CC=N1)CC1=CC=CC=N1
**1WQ**	0.222	C[C@@H]1OC2=CC=C(CN3CCC(=CC3)C3=CC=C(Cl)C=C3)C=C2NC1=O
**0MH**	0.222	COC1=CC=CC=C1C1=NOC(C)=C1C(=O)N1CCN(CC1)C1=CC(NC(=O)C2=CC=CS2)=C(C=C1Cl)[N+]([O-])=O
**A42**	0.221	COC1=CC(OC)=C(Cl)C=C1NC(=O)NC1=CN=C(C=N1)C#N
**5EZ**	0.221	ClC1=C(OC[C@@H]2CCNC2)C=C2C=CNC(=O)C2=C1
**NT5**	0.22	C[C@@H]1CC[C@@H](COC2=CC=C(F)C=N2)CN1C(=O)C1=CC(C)=CC=C1C1=NC=CC=N1
**K9Y**	0.22	CC1=CC=C(C=C1)N1N=C(C=C1NC(=O)NC1=CC=C(C=C1)N1C=NC2=C(N)N=CN=C12)C(C)(C)C
**JDS**	0.22	O\\N=C/C1=NC(CCCCNC2=C3C=C(Cl)C=CC3=NC3=C2CCCC3)=CC=C1O
**HWQ**	0.22	CN(CCC#N)C1=C2SC=CC2=NC(=N1)N(C)C1CCNCC1
**D5Z**	0.22	COC1=CC=C(C=C1OC)C1=NN(C2CCN(CC2)C2=C3C=CC=CC3=NC(N)=N2)C(=O)[C@@H]2CC=CC[C@H]12
**3MW**	0.22	NC1=CC=C(C=C1)C(=O)N[C@@H](C(=O)NO)C1=CC=C(C=C1)N1C=CC=N1
**WYE**	0.219	CN1C=C(\\C=C2/OC3=CC(O)=CC(O)=C3C2=O)C2=C(C=CN=C12)N1CCN(C)CC1
**SCQ**	0.219	BrC1=C2C=C(C=C(NCC3=CN=CN=C3)N2N=C1)C1=CC=CC=C1
**QXQ**	0.219	CCC(CC)(C=O)C(=O)NC1=CC=CC(CN2CCC3=CC4=C(OCO4)C=C3C2)=C1

**NTI**	0.219	CC(=O)OC1=CC=CC=C1C(=O)NC1=NC=C(S1)[N+]([O-])=O
**NJD**	0.219	COC1=NC(NCCC2=CNC3=CC=CC=C23)=NC(NC2=CC3=CNN=C3C=C2)=N1
**LTJ**	0.219	C\\C=C\\C(=O)N[C@H]1CCCN(C1)C1=C2C(C)=C(C)NC2=C(C=C1F)C(N)=O
**C9G**	0.219	CN1C=CC2=CC=C(N=C2C1=O)C1=CN(N=N1)C1=CC=C(O)C=C1
**5G3**	0.219	OC1=CC=C(C=C1)C(=N/C1=CC=CC=C1Cl)\\C1=CC=C(O)C=C1O
**5G4**	0.218	CC1=CC=CC=C1\\N=C(/C1=CC=C(O)C=C1)C1=CC=C(O)C=C1O
**30M**	0.218	CC(=O)NCCCCN1C2=C(C3=CC=CC=C13)C(=O)NCC2
**G9G**	0.217	CN1CCN(CC1)C1=NC2=C(NC(=O)C3=C2C=CC=C3)C=C1
